# Separation of iodate, bromide, nitrite, nitrate, and iodide in seawater by ion chromatography using 1-aminoundecyl group chemically bonded silica columns

**DOI:** 10.1007/s44211-024-00639-y

**Published:** 2024-08-14

**Authors:** Kazuaki Ito, Michinari Noguchi, Yuuta Horioka, Joji Ohshita, Takeshi Hirokawa

**Affiliations:** 1 Department of Biotechnology and Chemistry, Faculty of Engineering, Kindai University, 1 Umenobe, Takaya, Higashi-Hiroshima, Hiroshima 739-2116 Japan; 2 Cluster of Biotechnology and Chemistry system, Graduate School of System Engineering, Kindai University, 1 Umenobe, Takaya, Higashi-Hiroshima, Hiroshima 739-2116 Japan; 3 Present Address: Seawater Assessment Technologies Research Institute, 3-3-9 Yaga, Higashi-ku, Hiroshima 732-0042 Japan; 4 Smart Innovation Program, Graduate School of Advanced Science and Engineering, Hiroshima University, 1-4-1 Kagamiyama, Higashi-Hiroshima, Hiroshima 739-8527 Japan; 5 Division of Materials Model-Based Research, Digital Monozukuri (Manufacturing) Education and Research Center, Hiroshima University, 3-10-32 Kagamiyama, Higashi-Hiroshima, Hiroshima 739-0046 Japan; 6 Emeritus Professor, Hiroshima University, 1-4-1 Kagamiyama, Higashi-Hiroshima, Hiroshima 739-8527 Japan

**Keywords:** Ion chromatography, 1-Aminoundecyl group chemically bonded silica (AUS) stationary phase, High-capacity anion-exchange/hydrophilic/hydrophobic interaction mixed modes, NaCl mobile phase, Ultraviolet detection, Seawater, Inorganic anions (IO_3_^−^, Br^−^, NO_2_^−^, NO_3_^−^, and I^−^)

## Abstract

The separation and detection of six common inorganic anions (iodate (IO_3_^−^), bromate (BrO_3_^−^), bromide (Br^−^), nitrite (NO_2_^−^), nitrate (NO_3_^−^), and iodide (I^−^)) in pure water and 35 ‰ artificial seawater were examined by ion chromatography (IC). As packing materials of separation columns, 1-aminoundecyl group chemically bonded silica (AUS) gels were prepared. Separation of the anions in pure water was achieved using separation columns (150 mm × 4.6 mm i.d.) packed with the AUS gels, 0.1 M NaCl + 5 mM phosphate buffer (pH 4.5) as eluent, and a UV detector (wavelength 225 nm). The anions in artificial seawater were separated and detected with a 300 mm-long column without interferences by matrix anions such as chloride (Cl^−^) and sulfate (SO_4_^2−^). The stationary phases have high-capacity anion-exchange/hydrophilic/hydrophobic interaction mixed-modes. The IC system was applied to five inorganic anions, IO_3_^−^, Br^−^, NO_2_^−^, NO_3_^−^, and I^−^ in seawater of the Seto-Inland Sea, Japan. The detection limits (DLs, *S*/*N* = 3) were 11 µg L^−1^ (IO_3_^−^), 93 (Br^−^), 1.3 (NO_2_^−^), 1.4 (NO_3_^−^), and 1.1 (I^−^) for a 100-µL sample injection.

**Graphical abstract:**

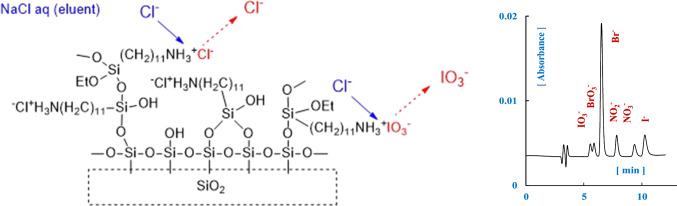

## Introduction

The choice of separation column and eluent for ion chromatography (IC) is critical for the analysis of minor anions, such as I^−^ [1], NO_2_^−^, and NO_3_^−^ [2], which are micronutrients in seawater [3]. On the other hand, Br^−^ which is relatively high in seawater have correlation with salinity [3, 4].

In our previous studies [4–7], the separation and UV (226 nm) detection of six anions, IO_3_^−^, BrO_3_^−^, Br^−^, NO_2_^−^, NO_3_^−^, and I^−^, in both pure water and 35 ‰ artificial seawater was achieved in a single run using octadecylsilane (ODS) and monolith ODS reversed-phase columns coated with *n*-dodecylammonium (DA^+^) and the eluent of 0.3 M NaCl + 5 mM phosphate buffer (pH 4.5). The columns preliminary coated with 5 mM DA^+^ worked as high-capacity anion-exchange columns. Low concentration of DA^+^ (0.5 mM) was added in the eluent to maintain both retention times of the anions and a stable baseline. These techniques are employed as the permanent coating method for ODS columns. Thus, simultaneous measurements of IO_3_^−^, Br^−^, NO_2_^−^, NO_3_^−^, and I^−^ were possible at intervals of 15–25 min per sample without interferences by salinity in seawater, while BrO_3_^−^ was not detected because of its low concentration [4–7], although BrO_3_^−^ is produced by oxidation of Br^−^ under the special conditions and known to be toxic [8]. Iodine (IO_3_^−^ and I^−^) and nitrogen (NO_2_^−^ and NO_3_^−^) are essential trace elements and nutrients in seawater, respectively [3, 9]. Br^−^ in seawater is relatively high in concentration and have high correlation with the salinity [3, 4].

The purpose of present study is the separation and UV detection of the six anions, IO_3_^−^, BrO_3_^−^, Br^−^, NO_2_^−^, NO_3_^−^, and I^−^ in seawater simultaneously using weakly basic anion-exchange columns. To the best of our knowledge, there are no results in seawater with any anion-exchange columns. In this study, simultaneous determination of the six anions in seawater was examined using high-capacity anion-exchange columns packed with 1-aminounndecyl group chemically-bonded silica (AUS) gels, which has higher potential stability than those of ODS coated with alkyl ammonium by hydrophobic interaction. The AUS gels were prepared by the reaction of silica gels with 11-aminoundecyl-triethoxysilane that is the longest aminoalkyltriethoxysilane commercially available.

The separation and UV detection of several inorganic anions, such as IO_3_^−^, Br^−^, NO_2_^−^, NO_3_^−^, and I^−^, was examined using protonated pyridine stationary phase bonded to silica gel and 15 mM Na_2_SO_4_ + 0.5 mM H_2_SO_4_ eluent (pH 3.3) by hydrophilic interaction chromatography (HIC) [10]. Similarly, HIC with UV detection has been examined to separate those anions on a triazole-functionalized anion exchanger with 5 mM Na_2_SO_4_ + 0.5 mM H_2_SO_4_ as eluent [11], a silica-based click lysine anion exchanger with 5 mM H_2_SO_4_ (pH 3.3) as eluent [12], and a poly (vinylimidazole-co-ethylene dimethacrylate) monolithic column with NH_4_Cl as eluent [13]. Retention behavior of inorganic anions by HIC was clearly described from the points of electrostatic interaction and partition [14]. However, those methods using low-capacity anion-exchange columns have been applied only to samples containing low salinity such as saliva, tap water, and river, lake water [9, 12, 13], but are not to saline solutions such as seawater.

## Experimental

### Reagents and methods

Unless otherwise specified, chemicals from Kanto Kagaku Co., Japan were used. Sodium salts of analytical grade were used for the preparation of standard anionic solutions and mobile phases. The standard anionic solutions were prepared by mixing or/and diluting the stock solutions of each anion (10 g L^−1^). Solutions of 0.1–0.5 M NaCl containing 5 mM phosphate buffer (pH 4.5) were used as eluents because good separation was obtained around of pH 4.5. The pH and buffer solutions were the same as those in the previous studies [4–7]. BET surface areas of silica gels were determined by nitrogen adsorption/desorption experiments. A membrane filter of mixed cellulose ester (pore size 0.45 μm, Advantec, Japan) was used to filtrate the eluents. Standard solutions of the six inorganic anions (10,000 mg L^−1^, each) were prepared by dissolving their sodium salts in ultrapure water. The composition table of Lyman and Fleming [15] was used as reference for the preparation of artificial seawater. Br^−^ was added as NaBr to prepare the samples. Anion-exchange capacities of stainless-steel columns (150 mm × 4.6 mm i.d.) were determined by a breakthrough method [11] using UV absorption of 5 mM NaNO_3_ in a flow rate of 0.5 ml/min.

### Preparation of packing materials and separation columns

**Fig. 1:**
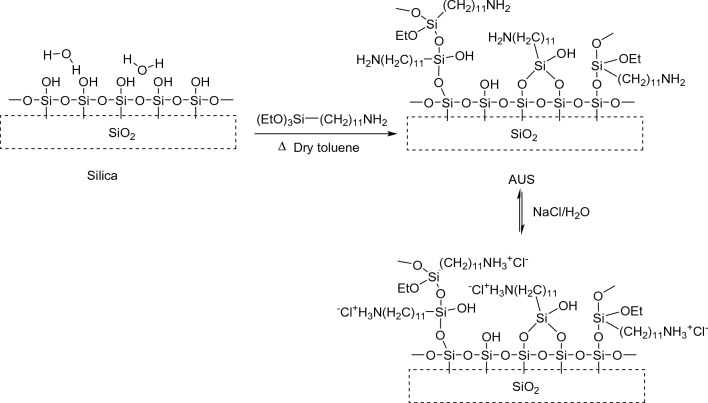
Expected reactions of silica and surface structures of the separation columns for anions

Two silica gels, Wakosil® 5SIL, 5 μm (BET surface area, 584 m^2^/g, Fujifilm Co. Japan) or Tosoh silica gel, 5 μm (BET surface area, 699 m^2^/g, Tosoh Co. Japan), and 11-aminoundecyltriethoxysilane (Gelest Co. USA) were added to dry toluene and refluxed at ca.111℃ under an argon atmosphere for 3 h while stirring, as is shown in Fig. 1. AUS gels obtained were dispersed by ultrasonication and collected by filtration. These silica-based stationary phases were further stirred in the mixed solvent of deionized water and methanol (50:50, v/v) for 2 h and then collected by filtration. The prepared stationary phases were packed in stainless-steel columns (150 mm × 4.6 mm i.d.) using the slurry method with a mixture of methanol and H_2_O mixture (50:50, v/v). The expected surface structures of AUS gels in acidic condition are also shown in Fig. 1.

### Measurement conditions and sample measurements

For anion separation, the eluents of 0.1–0.5 M NaCl + 5 mM phosphate buffer (pH 4.5) were used at a flow rate of 1 mL min^−1^. Measurements were performed at a wavelength of UV 225 nm with a sample injection volume of 100 µL. Seawater (coastal surface) samples in the Seto-Inland Sea (Hiroshima Bay, east side), which were filtered through a membrane filter (pore size, 0.45 µm), were injected directly without dilution.

## Results and discussion

### Anion separation in pure water and artificial seawater

**Fig. 2:**
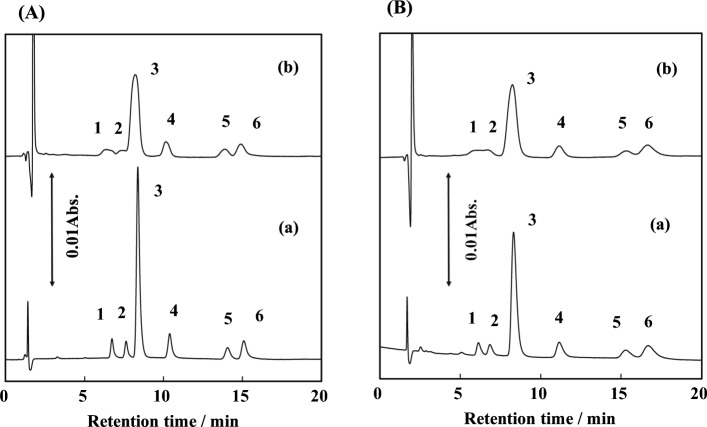
Ion chromatograms of anions in (a) pure water and (b) 35‰ artificial seawater. Column, 1-aminoundecyl group chemically bonded silica column (particle size, 5 μm; 150 × 4.6 mm i.d.) **A** Wako silica, **B** Tosoh silica; mobile phase, 0.1 M NaCl + 5 mM sodium phosphate buffer (pH 4.5); detection, UV 225 nm; flow rate, 1.0 mL min^−1^; sample volume, 100 μL. (1) IO_3_^−^ (0.5 mg L^−1^), (2) BrO_3_^−^ (1), (3) Br^−^ (50), (4) NO_2_^−^ (0.1), (5) NO_3_^−^ (0.1), (6) I^−^ (0.1)

Figure 2 shows the ion chromatograms of the six anions (IO_3_^−^, BrO_3_^−^, Br^−^, NO_2_^−^, NO_3_^−^, and I^−^) in pure water and artificial seawater using two types of silica columns containing 1-aminoundecyl groups and NaCl (0.1 M) eluent in the acidic (pH 4.5) condition. In Fig. 2A(a) with Column 1 (silica gel, Wakosil® 5 SIL), the six anions in pure water were successfully separated within 16 min in the same order of elution as those observed with the DA^+^-coated ODS/monolith ODS columns with proper separation [4–7]. The results contrasted the case of dilauryldimethylammonium (DDA^+^)-coated ODS column [16], where DDA^+^ has two methyl and two lauryl groups (longer CH_2_ chain), leading to a shorter retention time for IO_3_^−^ and a longer retention time for I^−^, owing to the lack of hydrophilic interaction and the strong hydrophobic interaction, respectively. Compared to Fig. 2A(a), the peak widths in artificial seawater (Fig. 2A(b)) were broadened for all the anions. This salinity effect was pronounced for hydrophilic ions IO_3_^−^ and BrO_3_^−^, because the matrix ions [i.e., high concentrations of Cl^−^ (0.56 M) and SO_4_^2−^ (0.03 M) in artificial seawater] inhibited the retention of analytes near the inlet of the separation column.

Similar separation patterns were also observed for Column 2 (silica gel 5 μm, Tosoh), as shown in Fig. 2B(a) and (b). Thus, there were no differences between the two columns, Columns 1 and 2 in terms of separation order for the anions and also characteristics of matrix effects by salinity observed in pure water and artificial seawater.

**Fig. 3:**
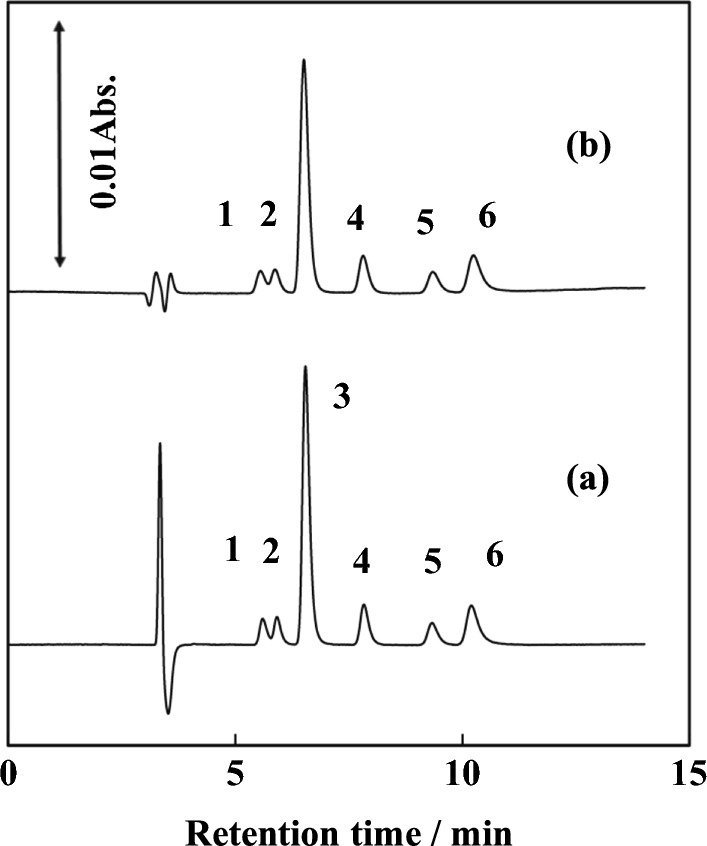
Ion chromatograms of anions in (a) pure water and (b) 35‰ artificial seawater. Column, 1-aminoundecyl group chemically bonded silica column (particle size, 5 μm; 300 × 4.6 mm i.d.); mobile phase, 0.5 M NaCl + 5 mM sodium phosphate buffer (pH 4.5). Other conditions were the same as in Fig. 3

Figure 3 shows the ion chromatograms of the six anions in pure water and artificial seawater using a 300 mm-long column connecting Columns 1 and 2 described above. A high-concentration (0.5 M) of NaCl eluent containing 5 mM phosphate buffer (pH 4.5) was used. Anion separation was achieved in a short period without interferences despite the large amounts of coexisting anions in artificial seawater.

In the preparation of the packing materials, the ratios (A/S) of 11-aminoundecyltriethoxysilane (A, unit g) and silica gel (S, unit g) were 0.26 and 0.70, respectively, (see “Preparation of packing materials and separation columns” in the Experimental section). The anion-exchange capacities of Columns 1 and 2 were 0.51 and 0.63 meq./column (150 mm × 4.6 mm i.d.), respectively. No large difference in anion-exchange capacities was apparent between the two columns in spite of large difference in A/S. The anion-exchange capacities in our study were ca. (1.9 and 2.4) × 10^2^ times higher, respectively, compared with the silica column containing click lysine anion exchanger (0.00267 meq./column, 150 mm × 4.6 mm i.d.), where IO_3_^−^, BrO_3_^−^, Br^−^, NO_3_^−^, I^−^, and SCN^−^ were eluted in ca. 2.6–4.8 min with 5 mM Na_2_SO_4_ (pH 4.4) eluent at a flow rate of 1 mL/min. These results suggest that the most surfaces of two silica gels in our studies would be covered with 1-aminoundecyl groups.

### Separation characteristics

**Fig. 4:**
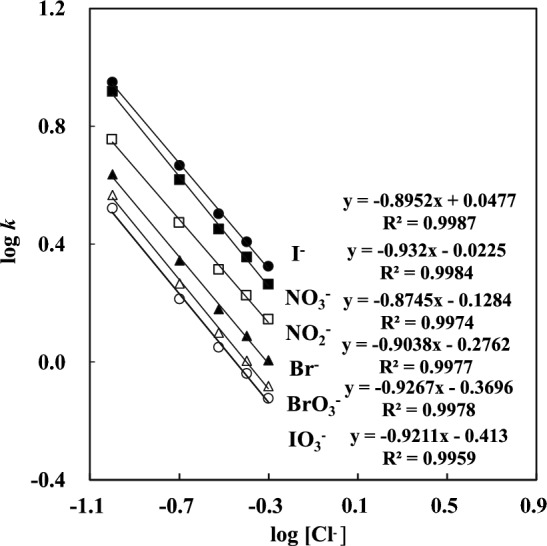
Logarithm of the retention factor (log *k*) for anions in artificial seawater as a function of the logarithm of the eluent concentration. IC conditions were the same as in Fig. 3

Figure 4 shows the correlation between the retention capacity for the six anions in 35 ‰ artificial seawater and the concentration of the eluent. Columns 1 and 2 (300 mm in length) were used. The slopes for the six anions were in the range of −0.87 (NO_2_^−^) to −0.93 (NO_3_^−^) and were almost the same as −0.88 (for NO_2_^−^) to −0.93 (for NO_3_^−^) in pure water. This suggests that the interferences by salinity in the artificial seawater were minor in this study. The separation column with high anion-exchange capacity (approximately 1.14 meq./300 mm) worked well for anion separation without interferences by the salinity in artificial seawater. Three kinds of interactions, high-capacity anion-exchange/hydrophilic interaction/hydrophobic interaction, seem to operate to enable the separation of target ions in samples containing a large amount of salinity, similarly to those suggested for DA^+^-coated ODS and monolith ODS columns. The reason for high anion-exchange capacity is not clear yet. However, high loading of ammonium units by chemical bonding seems to be responsible.

### Application to seawater samples

**Fig. 5:**
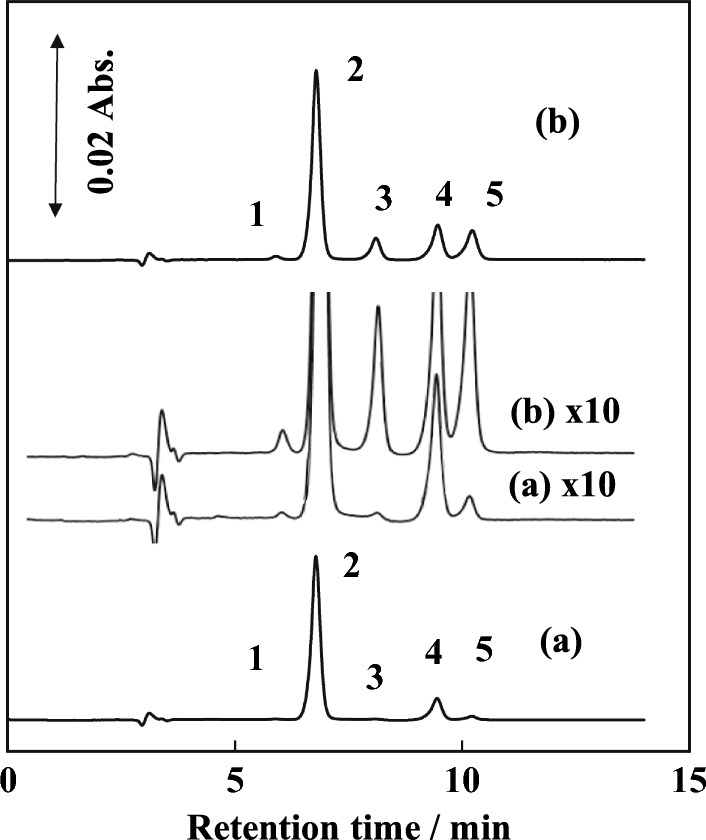
Ion chromatograms of seawater samples. (a) Surface seawater and (b) the seawater spiked with 0.1 mg/L of IO_3_^−^, NO_2_^−^ NO_3_^−^, I^−^, and 10 mg/L of Br^−^. (1) IO_3_^−^, (2) Br^−^, (3) NO_2_^−^, (4) NO_3_^−^, (5) I^−^. IC conditions were the same as in Fig. 3

The ion chromatograms of seawater and the spiked seawater samples are shown in Fig. 5. A 300 mm-long column was used (anion-exchange capacity: 1.3 meq./300 mm). Coastal surface seawater samples from the Seto-Inland Sea, Japan (Hiroshima Bay, east side) without and with five anions (final concentration: 0.1 mg L^−1^ for IO_3_^−^, NO_2_^−^, NO_3_^−^, and I^−^ and 10 mg L^−1^ for Br^−^) were directly injected without sample dilution after permeation by membrane filter (0.5 µm: porosity). The results obtained for a seawater sample were 24 µg L^−1^ (IO_3_^−^), 54.5 mg L^−1^ (Br^−^), 3 µg L^−1^ (NO_2_^−^), 0.12 mg L^−1^ (NO_3_^−^), and 15 µg L^−1^ (I^−^). The recovery rates were in the range of 95–104%. The detection limits (DLs, *S*/*N* = 3) were 11 µg L^−1^ (IO_3_^−^), 93 µg L^−1^ (Br^−^), 1.3 µg L^−1^ (NO_2_^−^), 1.4 µg L^−1^ (NO_3_^−^), and 1.1 µg L^−1^ (I^−^) for a 100-µL sample injection.

## Conclusion

Silica-based stationary phases with chemically bonded primary amine groups (1-aminoundecyl group) which had high anion-exchange capacities were prepared for the determination of five anions, IO_3_^−^, Br^−^, NO_2_^−^, NO_3_^−^, and I^−^, in seawater. The columns have high-capacity anion-exchange/hydrophilic/hydrophobic interaction mixed-mode stationary phases. Seawater samples were directly injected without sample dilution after filtration. The separation and UV determination of the five anions in seawater was performed in shorter times without interferences by large amounts of coexisting anions, such as chloride and sulfate. Although the detection limits of anions are not sufficiently low for accurate quantification, the simple sample processing by the present system without dilution/concentration is useful and practical.
